# SHANK family on stem cell fate and development

**DOI:** 10.1038/s41419-022-05325-3

**Published:** 2022-10-18

**Authors:** Xu Liu, Mengmeng Yuan, Benson Wui-Man Lau, Yue Li

**Affiliations:** 1 State Key Laboratory of Component-Based Chinese Medicine, Institute of Traditional Chinese Medicine, Tianjin University of Traditional Chinese Medicine, Tianjin, China; 2 Department of Rehabilitation Sciences, The Hong Kong Polytechnic University, Hong Kong SAR, China

**Keywords:** Stem cells, Cell biology

## Abstract

SH3 and multiple ankyrin repeat domains protein (SHANK) 1, SHANK2, and SHANK3 encode a family of postsynaptic scaffolding proteins present at glutamatergic synapses and play a crucial role in synaptogenesis. In the past years, studies have provided a preliminary appreciation and understanding of the influence of the SHANK family in controlling stem cell fate. Here, we review the modulation of SHANK gene expression and their related signaling pathways, allowing for an in-depth understanding of the role of SHANK in stem cells. Besides, their role in governing stem cell self-renewal, proliferation, differentiation, apoptosis, and metabolism are explored in neural stem cells (NSCs), stem cells from apical papilla (SCAPs), and induced pluripotent stem cells (iPSCs). Moreover, iPSCs and embryonic stem cells (ESCs) have been utilized as model systems for analyzing their functions in terms of neuronal development. SHANK-mediated stem cell fate determination is an intricate and multifactorial process. This study aims to achieve a better understanding of the role of SHANK in these processes and their clinical applications, thereby advancing the field of stem cell therapy.

**Figure:**
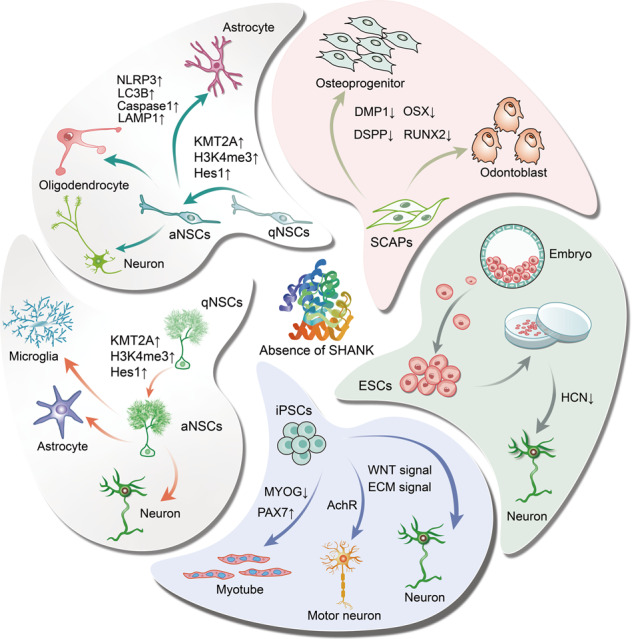
This review unravels the regulatory role of the SHANK family in the fate of stem cells.

This review unravels the regulatory role of the SHANK family in the fate of stem cells.

## Facts

- SHANK family proteins interact with various molecules through functional domains.
- SHANK gene expression is governed by various factors hinging on the cellular and organismal context.
- SHANK family proteins directly or indirectly govern the expression of a remarkably large number of genes.
- Molecular mechanisms underlying the regulation of the SHANK family in fate determination of diverse stem cell populations, such as SCAPs, NSCs, iPSCs, and ESCs, are still not fully illustrated yet.
- SHANK family plays a crucial role in stem cell self-renewal, proliferation, differentiation, apoptosis, and metabolism.

## Open questions

- What is the challenge of stem cell therapy and its recent advances?
- How SHANK family governs stem cell fate?
- How to balance the SHANK family and interacting proteins or downstream targets and their wane and wax?
- What is the cellular and molecular mechanism underlying SHANK family regulation of NSCs, neurogenesis and niche responses to physiological and pathological stimuli?
- What’s the prospect of restoring SHANK family expression in a clinical application?

## Introduction

**Fig. 1:**
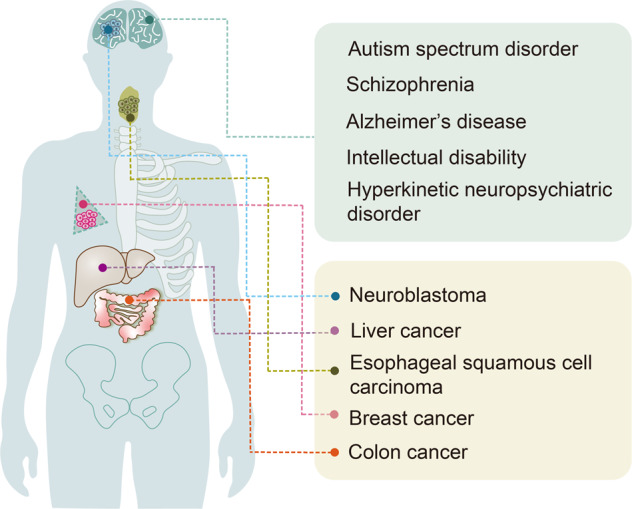
Schematic diagram of diseases caused by SHANK mutations. Mutations of SHANK have been identified in many human diseases, including neurodevelopmental disorders (highlighted in green box) and cancers (highlighted in yellow box).

SHANK proteins are master scaffolding proteins that tether and organize intermediate scaffolding proteins located at the postsynaptic density of glutamatergic synapses that regulate the synaptic formation, development, and plasticity [1, 2]. SHANK family proteins are encoded by three genes, namely *Shank1*, *Shank2*, and *Shank3*. To begin, observations highlighting the importance of SHANK in human health have been made in the context of Phelan McDermid syndrome (PMS), and genetic studies have identified SHANK3 haploinsufficiency as the cause of the disease [3–5]. SHANK family genes mutations have been closely associated with neurodevelopmental disorders, including autism spectrum disorder (ASD), intellectual disability (ID), schizophrenia (SCZ), and Alzheimer’s disease (AD) (Fig. 1) [6]. Furthermore, *SHANK3* overexpression results in a hyperkinetic neuropsychiatric disorder [7]. Interestingly, a subset of human epithelial cancers exhibits focal amplification of SHANK genes (Fig. 1). Among them, SHANK1 and SHANK2 have been found to be potential oncogenes whose overexpression promotes cellular transformation and tumor formation [8, 9]. In parallel, mutations in the SHANK family genes generate alterations in diverse intracellular signaling pathways and physiological and pathological processes such as cell fate determination, stem cell regulation, neural maturation, and cancer [6, 10, 11]. Currently, considerable progress has been made in establishing a link between SHANK and mature neurons, exposing that the former plays a pivotal role in stem cell fate determination. Stem cells are usually categorized into pluripotent and adult stem cells, enabling self-renewal, proliferation, and multi-directional differentiation, which are crucial for development, tissue homeostasis, and injury repair [12–14]. Stem cells have enormous potential in medicine owing to their unique properties, such as stem cell transplantation therapy, which has been successfully used in clinics to alleviate patients’ symptoms [15]. Thus, the primary objective of this review is to explore the relationship between SHANK and stem cells in order to provide exciting novel therapeutic options for *Shank*-related diseases.

This review aims to summarize current knowledge and provide an overview of the SHANK family and stem cell biology (Graphical abstract). Herein, we review the structure and function of the SHANK family, describe the regulatory mechanism of the SHANK family, and discuss the management of signaling pathways to elucidate the function of each domain and its signal transduction network. Afterward, we highlight the roles of SHANK in stem cells, including SCAPs, NSCs, iPSCs, and ESCs, and further outline the implications of these findings in terms of their role in the development, regeneration, and advancements in disease therapy.

## Shank family

### SHANK family structure and function

**Fig. 2:**
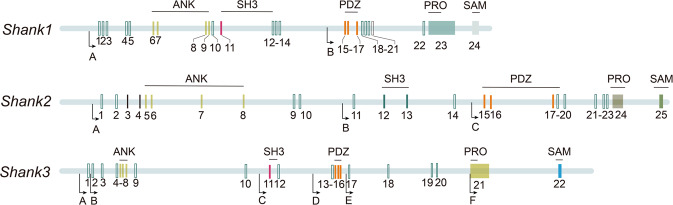
Schematic diagram of SHANK genes: structure and intragenic expressed region. The schematic diagram shows the full structure of the SHANK genes and the location of intragenic promoters within these genes. The location of the protein domains is included above their respective encoding exons (in the figure, exons are numbered).

**Fig. 3:**
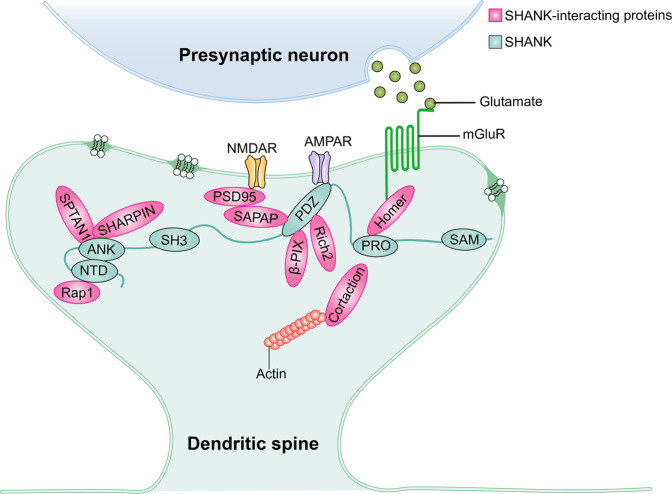
Schematic diagram of SHANK-interacting proteins at the postsynaptic site. A representation of SHANK located in the PSD is shown, with its protein–protein interaction domains illustrated.

SHANK genes code for scaffold proteins located at excitatory synapses and are encoded by the *Shank1*, *Shank2*, and *Shank3* genes. The *Shank3* gene is positioned on mouse chromosome 15E3 (human location: 22q13.3), containing 22 exons, and spans 60 kilobases of genomic DNA. It is widely accepted as the most complex of the three SHANK gene members owing to the presence of six intragenic promoters that can code for several mRNA splice variants, resulting in the possible generation of several protein isoforms [1]. Moreover, SHANK3 contains five domains that form the basis for protein–protein interactions: ankyrin repeat domain (ANK), SRC homology 3 (SH3) domain superfamily, postsynaptic density protein 95 (PSD95)-disks large homolog 1-zonula occludens 1 (PDZ) domain, proline­rich region (PRO), and sterile alpha motif (SAM) (Fig. 2). Based on these domains, SHANK3 is known to interact with several synaptic proteins, including other scaffold molecules, glutamatergic receptors, signaling, and cytoskeletal proteins (Fig. 3). The ANK domain interacts with the PSD protein SHARPIN and probably binds to the cytoskeleton through interactions with the spectrin alpha chain, non-erythrocytic 1 (SPTAN1). Furthermore, the ANK domain couples with the N-terminal domain (NTD) to form an NTD-ANK supra module [16], which binds to two copies of Rap1 with distinct modes, and prevents Rap1 guanosine triphosphate (GTP) hydrolysis catalyzed by SynGAP [17]. However, this intramolecular interaction presumably limits access to binding partners such as SHARPIN and SPTAN1 [18]. The PDZ domain interacts with NMDA-type glutamate receptors by GKAP/SAPAPs, which bind the PSD95–NMDAR complex [19] and the glutamate receptor 1 (GluR1) subunit of AMPA receptors (AMPARs), which is crucial for dendritic spine formation and synaptic transmission [20]. Metabotropic glutamate receptor (mGluR) is linked to the SHANK3 protein complex by Homer proteins that bind to the PRO domain and can also bind to cortactin, which is highly implicated in cytoskeleton regulation, synaptic transmission, and plasticity [19, 21]. In addition, the carboxy­terminal SAM domain is involved in SHANK multimerization and is required for the localization of SHANK3 to PSD [19, 22, 23].

In contrast, the *Shank2* gene is located on mouse chromosome 7F5 (human location: 11q13.2), which has 25 exons and spans about 450 kb of mouse genomic DNA. Compared to six intragenic promoters in *Shank3*, *Shank2* possesses three alternative promoters, resulting in distinct protein isoforms. Three isoforms of SHANK2 are produced that contain SHANK2E (containing all five protein domains), SHANK2A (lacking ANK), and SHANK2C (lacking ANK and the SH3 domain). Besides, another isoform of SHANK2-SHANK2B is transcribed from the same intragenic promoter as SHANK2A but exhibits alternative splicing.

Lastly, *Shank1* is located on mouse chromosome 7B4 (human location: 19q13.33), which has 24 exons and spans about 50 kb of genomic DNA. It has two isoforms that are generated by different promoters. In addition, *Shank1* also contains alternative splicing sites that can generate different transcripts [24]. Here, the three SHANK genes can codify several mRNA splice variants that generate multiple protein isoforms [25].

Similarly, SHANK2 and SHANK1 share functional domains with SHANK3 containing ANK, SH3, PDZ, PRO, and SAM (Fig. 2), which interact with multiple partners at the synapse (Fig. 3) [2, 19, 26], but may also possess distinct functions. For example, SAM domain self-multimerization is necessary for the localization of SHANK3 and SHANK2 to PSD, but not required for SHANK1. The localization of SHANK1 to synapses is mediated by the PDZ domain binding to GKAP/SAPAP [27, 28]. Evidently, SHANK family proteins interact with various molecules through functional domains, influencing the expression of both themselves and their partners. Therefore, understanding the function of each protein domain, the regulatory factors of SHANK, and the direction of the signaling they mediate will provide invaluable insights for determining the pathogenicity of human mutations.

### Regulation of SHANK family

SHANK gene expression is most likely governed at different levels by various factors hinging on the cellular and organismal context. The expression of mammalian *Shank1*, *Shank2*, and *Shank3* may be regulated differentially to some extent. *Shank1* mRNA is abundant in the hippocampus (CA1 and CA3), cortex, amygdala, and cerebellar Purkinje cells and is the only family member present in the hypothalamus, whereas low levels of *Shank1* have been identified in the striatum [24, 29–31]. *Shank2* mRNA is also highly expressed in the brain. In particular, *Shank2* mRNA is present in the hippocampus (CA1 and CA3), dentate gyrus, striatum, cortex, olfactory bulb, and cerebellum and is selectively expressed only in Purkinje cells [24, 31]. Moreover, it is expressed in the kidney and liver at lower levels. *Shank3* mRNA is highly expressed in the heart and moderately expressed in the brain and spleen [24]. In the brain, *Shank3* mRNA expression overlaps with *Shank2* mRNA distribution except in the cerebellum, where *Shank3* expression is restricted to granule cells [32–34]. Additionally, the *Shank3* expression level is increased in CA3 compared to CA1.

SHANK family expression may be controlled by diverse inputs. During the brain development of mice, S100β can negatively modulate SHANK2 and SHANK3 levels in a zinc-dependent manner to affect synaptic function and maturation [35]. Another factor that may trigger fluctuations in SHANK gene expression is sex hormones; both dihydrotestosterone and 17β-Estradiol can elevate the expression of SHANK genes [36]. At the epigenetic level, DNA methylation and histone acetylation can cause tissue-specific expression of different SHANK3 isoforms [6, 37, 38]. Studies also have validated kinases, including ribosomal S6 kinase (RSK) 2, extracellular signal-regulated kinase (ERK) 2, Ca^2+^/calmodulin dependent protein kinase (CaMKII), and ubiquitin-specific proteases (USP) 8, participate in the regulation of SHANK expression [39]. The first report about SHANK being phosphorylated by kinases indicated that RSK2 can phosphorylate SHANK1 and SHANK3 to regulate synaptic transmission [40]. Likewise, CaMKII phosphorylates SHANK3 S782 to influence synaptic properties [41]. Furthermore, ERK2 can promote SHANK3 poly-ubiquitination-dependent degradation by binding to it and phosphorylating it at three residues to modulate its stability and activity [39]. USP8, as a deubiquitinating enzyme, also can enhance SHANK3 and SHANK1 protein levels to elevate dendritic spine density [42].

Regulation of SHANK gene expression at the post-transcriptional level has also been reported. MicroRNAs (miRNAs) are small non-coding RNAs and, as critical post-transcriptional regulators, downregulate mRNA expression by lowering mRNA stability or inhibiting translation through binding to the 3′ untranslated regions (3′UTRs) of target mRNAs [43]. miR-7, miR-34a, and miR-504 can modulate *Shank3* mRNA but not *Shank1* and *Shank2* [44]. Likewise, *Shank3* mRNA expression may also be mediated by miR-873 [45]. miR-137 regulates *Shank2* expression by repressing protein translation rather than inducing mRNA degradation [46].

Given these disparate data, there is an urgent need for a more integrated and systematic analysis of the mechanism by which SHANK expression is controlled. This will contribute to a better understanding of the regulation and development of SHANK in stem cells.

### Regulation of signaling pathways by the SHANK family

**Fig. 4:**
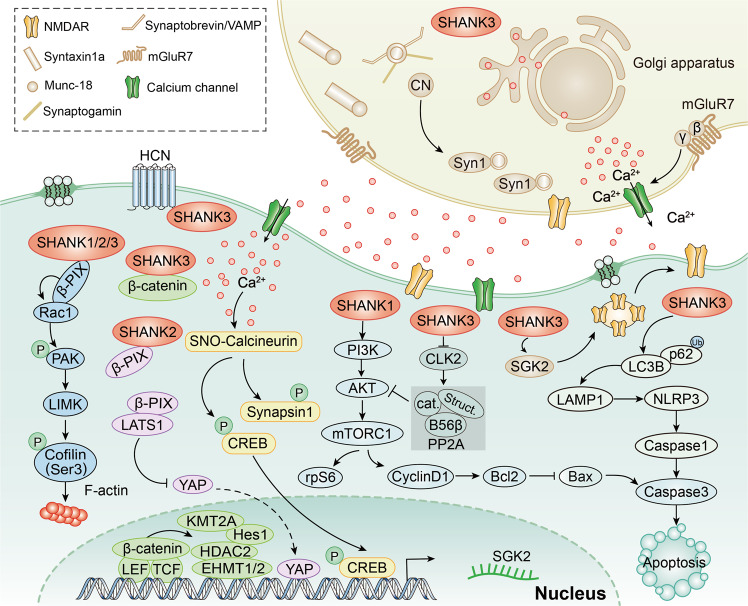
Schematic diagram of SHANK in the management of signaling pathways. SHANK3 can interact with HCN proteins and also form a complex with β-PIX, which regulates the activity of Rac1/PAK and Cofilin. Furthermore, SHANK3 can bind to β-catenin to regulate HDAC2, EHMT1/2 and KMT2A. In addition, SHANK3 can influence the balance of Ca^2+^ homeostasis, thereby resulting in changes in the phosphorylation of Synapsin1 and CREB. SHANK3 also can modulate the expression of SGK2 and the expressions of several genes associated with autophagy and inflammation, including LC3B, LAMP1, NLRP3 and Caspase1. SHANK3 can influence the phosphorylation and activation of PP2A regulatory subunit-B56β and regulation of mTORC1 signaling by regulating CLK2 expression. SHANK2 can regulate the expression of YAP through modulating the interaction between β-PIX and LATS1. SHANK1 can regulate AKT/mTOR to modulate the expression of Bcl2, Bax and Caspase3.

Mutations in functional SHANK proteins cause diverse signal pathway alterations both in patients and animal models (Fig. 4). SHANK family proteins directly or indirectly govern the expression of a remarkably large number of genes (Table 1), indicating their significant role in gene regulation. SHANK3 can typically bind to β-catenin; when the absence of SHANK3 results in the translocation of β-catenin from synapses into the nucleus, where it can form complexes with transcription factor TCF/LEF to activate target genes such as histone deacetylase (HDAC) 2, euchromatic histone-lysine N-methyltransferase (EHMT) 1/2, and histone-lysine methyltransferase 2 A (KMT2A), thereby impacting synaptic function and stem cell behavior [47–49]. Furthermore, earlier studies have exposed that SHANK forms a complex with Rho guanine nucleotide exchange factor 7 (β-PIX) [50] that regulates Rac1/PAK/Confilin activity to modulate cortical actin filaments and influences NMDAR synaptic function [51]. SHANK3 also interacts with hyperpolarization-activated cyclic nucleotide-gated channel (HCN) proteins, forming I_h_-channels to regulate neuronal morphology and synaptic connectivity [52]. Additionally, numerous intracellular signaling pathways are regulated. However, the role of SHANK in the direct regulation of these protein expressions remains elusive. SHANK3 deficiency results in the upregulation of Cdc2­like kinase 2 (CLK2) expression, then lead to the phosphorylation and activation of serine/threonine protein phosphatase 2A (PP2A) regulatory subunit-B56β and eventually the down-regulation of protein kinase B (PKB/AKT)-mammalian target of rapamycin complex 1 (mTORC1) signaling to modulate synaptic function [53]. Moreover, SHANK3 deficiency induces endosomal/lysosomal and ubiquitin aggregation together with mitochondrial impairment and inflammasome activation, including an increase in microtubule-associated protein 1 light chain 3 beta (LC3B) and lysosomal-associated membrane protein 1 (LAMP1) levels, and the activation of NLR family pyrin domain containing 3 (NLRP3) and Caspase1, all of which act to modulate NSC and astroglial metabolism [10]. In the absence of SHANK3, the expression of serum- and glucocorticoid-inducible kinase 2 (SGK2) is downregulated, and NMDAR function is impaired [54]. Additionally, a mutation in the SHANK3 may lead to an imbalance in Ca^2+^ homeostasis, which is responsible for intracellular nitric oxide (NO) production, thereby resulting in the S-nitrosylation (SNO) of many proteins. SNO-Calcineurin increases the phosphorylation of Synapsin1 and cAMP-response element binding protein (CREB), affecting synaptic vesicle mobilization and gene transcription, respectively [55]. Recently, SHANK1 and SHANK2 have been found as potential oncogenes. On the one hand, the former can trigger AKT/mTOR signaling and the mitochondrial apoptotic pathway to modulate cell proliferation, migration, and apoptosis [9]. On the other hand, the latter regulates Hippo signaling, and its overexpression leads to YAP activation and cellular transformation [8].

**Table 1:** Signaling pathways regulation by the SHANK family.

Protein	Species	Mechanisms	Phenotypes	References
SHANK3	Mouse	β-catenin, HDAC2	Transcriptional activity	[47]
SHANK3	Mouse	β-catenin, EHMT1/2	NMDAR synaptic function	[48]
SHANK3	Mouse	β-catenin, KMT2A, Hes1	NSC proliferation and differentiation	[49]
SHANK1/SHANK2/SHANK3	Mouse/Rat/ Human	β-PIX, Rac1/PAK/ /Cofilin	NMDAR synaptic function, actin regulation	[50, 51]
SHANK3	Human	HCN	Neuronal morphology and synaptic connectivity	[52]
SHANK3	Mouse	CLK2, PP2A, B56β, AKT	Synaptic function	[53]
SHANK3	Mouse	LC3B, LAMP1, NLRP3, Caspase1	NSC and astroglial metabolism	[10]
SHANK3	Mouse	SGK2	Vesicle mobilization, gene transcription, and synaptic function	[54]
SHANK3	Mouse	SNO-proteome, Synapsin1, CREB	NMDAR synaptic function	[55]
SHANK2	Human/Mouse	Hippo	Cell proliferation and tumor formation	[8]
SHANK1	Human	Bax, Bcl2, Caspase3, and AKT/mTOR	Cell proliferation, migration, and apoptosis	[9]

Collectively, these studies provide compelling evidence that SHANK plays a causal role in modulating signaling pathways, and most of them play a crucial but sometimes disparate role in various types of stem cells. Taken together, the multitudinous genes and processes regulated by the SHANK family position them high in the hierarchy of gene network regulatory mechanisms that control stem cell identity and behavior in a variety of contexts, which suggests the stem cell model can be utilized to seek therapeutic targets.

## Shank and stem cells

Stem cells exhibit two defining characteristics, the capacity for self-renewal through cell division and that for generating specialized cell type(s) through differentiation, which contributes significantly to organ homeostasis and regeneration [12–14]. There are two main types of stem cells: embryonic stem cells and adult or tissue stem cells, derived from the inner mass of blastocysts and various tissues, respectively [13]. Stem cell behaviors are strongly influenced by extrinsic regulators and intrinsic factors, some of which are common for all stem cells, while others are unique to certain stem cell types [14, 56]. Therefore, uncovering the regulation of stem cells is critical not only for their therapeutic potential but also for providing more opportunities to explore mechanisms for the treatment of diseases.

At present, SHANK deficiency is largely considered a deficit in mature neurons, so SHANK functions have been investigated primarily in neurons and synapses. However, some new studies have reported that SHANK may also play a critical role in several types of stem cells (Table 2). It is important to gain a deeper understanding of the role of SHANK in the regulation of stem cells. On the one hand, the gene expression regulated by SHANK plays a key role in the application of stem cell therapy for providing essential information. On the other hand, stem cells facilitate the exploration of novel mechanisms and potential treatments for *Shank*-related diseases.

**Table 2:** The role of the SHANK family in regulating stem cell behaviors.

Protein	Cell types	Species	Phenotypes	Mechanisms	References
SHANK2	SCAPs	Human	Osteo/dentinogenic differentiation	DSPP, DMP1, RUNX2, and OSX	[60]
SHANK3	NSCs	Mouse	Proliferation and differentiation	N/A	[11]
SHANK3	NSCs	Mouse	Self-renewal, proliferation, differentiation, apoptosis, and metabolism	Caspase3, LC3B, LAMP1, NLRP3, Caspase1	[10]
SHANK3	NSCs/iPSCs	Mouse/human	Proliferation and differentiation	β-catenin, KMT2A, Hes1	[49]
SHANK3	iPSCs	Human	Transcription, neural differentiation	Wnt signal and ECM signal	[70]
SHANK3	iPSCs	Human	Neuronal differentiation	N/A	[71]
SHANK3	iPSCs	Human	Neuronal differentiation and maturation, myogenic cells maturation	AChRs, PAX7, and MYOG	[73]

### SHANK and SCAPs

**Fig. 5:**
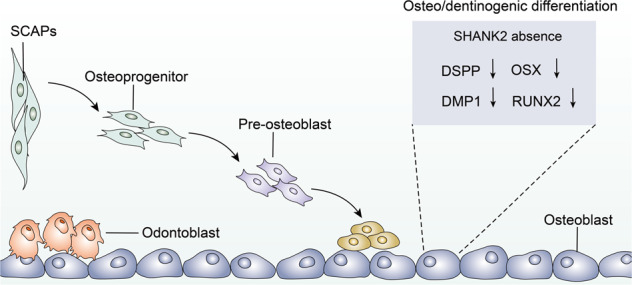
Schematic diagram representing the effects of SHANK2 on the differentiation of SCAPs. SHANK2 regulates osteo/dentinogenic differentiation in SCAPs via DSPP, DMP1, OSX and RUNX2.

SCAPs are a unique postpartum stem cell population residing in the apical papilla of immature permanent teeth, representing a new type of dental mesenchymal stem cells (MSCs) population that possess self-renewal, proliferation, and multilineage differentiation properties [57, 58]. Their plasticity allows them to differentiate into multiple cell lineages, including osteoblasts, odontoblasts, neural cells, adipocytes, chondrocytes, and hepatocytes [58]. Due to their low immunogenicity, SCAPs have the potential for tissue regeneration and repair in several areas and are considered an attractive alternative cell source for stem cell-based therapies [59]. Therefore, elucidating the processes and mechanisms underlying the control of SCAP fates could be an effective strategy for clinical application. Notably, SHANK2 exerts a peculiar role in determining the fate of SCAPs [60]. Upon examining the role of SHANK2 in dental tissue-derived MSCs, SHANK2 is proven to highly express in differentiated dental tissue-derived MSCs such as periodontal ligament stem cells (PDLSCs), dental pulp stem cells (DPSCs), and SCAPs compared to MSCs of other origins, such as BMSCs, Wharton’s Jelly of the umbilical cord (WJCMSCs) and adipose-derived mesenchymal stem cells (ADSCs) [60]. Among them, SCAPs have the ability to differentiate into odontoblast-like cells, and the cell population expresses high levels of survivin and telomerase, which are both pivotal molecules in mediating cell proliferation [60]. When SHANK2 is silenced in SCAPs, alkaline phosphatase (ALP) activity is inhibited, which is a specific marker of early osteogenic differentiation [60]. Mineralization is also decreased, and the expressions of key osteo/dentinogenic transcription factors (RUNX2 and OSX) and dentinogenic markers (DSPP and DMP1) are repressed (Fig. 5). However, osteoblast-related genes such as BSP, OPN, and OCN remain unchanged [60], indicating that SHANK2 may participate in the differentiation of SCAPs mainly through odontoblast lineages rather than osteoblast lineages. These findings reveal that SHANK2 is highly implicated in regulating SCAPs differentiation, while its role in the fate regulation of SCAPs multilineage differentiation warrants further investigation. Thus, it will offer opportunities to specifically promote cell differentiation into a lineage of interest, which may provide a new strategy for the clinical application of SCAPs in tissue regeneration and repair, including bone regeneration, pulp-dentin regeneration, neural regeneration, and periodontal tissue regeneration.

### SHANK and NSCs

NSCs are a group of stem cells of the adult central nervous system (CNS) that exhibit self-renewal and multipotency to support neural cells generation, including neurons, astrocytes, or oligodendrocytes in a regional and developmental stage-appropriate manner [61–63]. They persist and remain active in two specific “neurogenic” brain regions: the subgranular zone (SGZ) in the dentate gyrus of the hippocampus and the subventricular zone (SVZ) of the lateral ventricles [64]. Aberrant development of NSCs can induce various neurological diseases, such as fragile X syndrome (FXS), ASD, AD, and so forth. SHANK family proteins are critical to determine the fate of NSCs. Consequently, the process by which SHANK regulates the development of NSCs is a topic worthy of investigation. Few studies have explored the relationship between SHANK and NSC fate specification. Indeed, exploring the fate regulation of NSCs is essential to understanding how mammalian brains develop and whether these endogenous NSCs and progenitor cells can be activated for the neural repair of diseases and injuries.

**Fig. 6:**
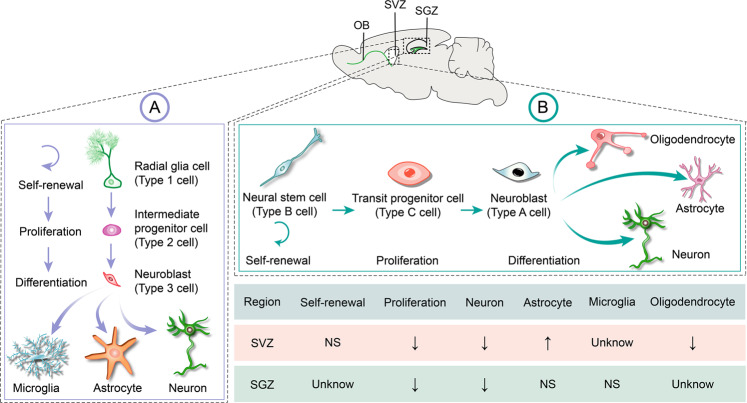
Schematic diagram of the influence of SHANK3 deficiency in determining the fate of NSCs. **A** The influence of SHANK3 on NSC proliferation and differentiation in SGZ. **B** The influence of SHANK3 on NSC self-renewal, proliferation, and differentiation in SVZ.

**Fig. 7:**
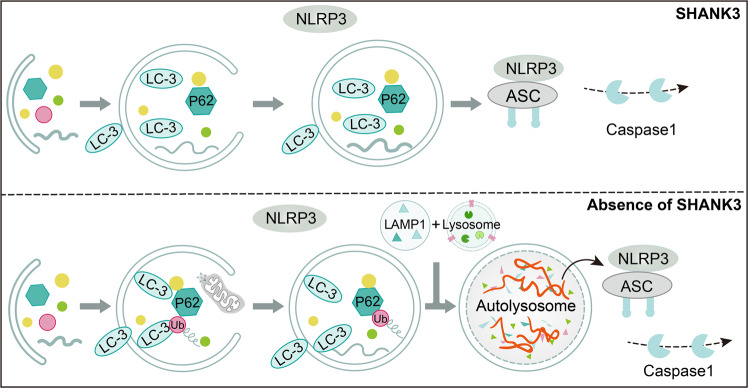
Schematic diagram representing the effects of SHANK3 on the metabolism of NSCs and astrocytes. SHANK3 regulates the metabolism of NSCs and astrocytes through modulating the expressions of LC3B, LAMP1, NLRP3 and Caspase1.

**Fig. 8:**
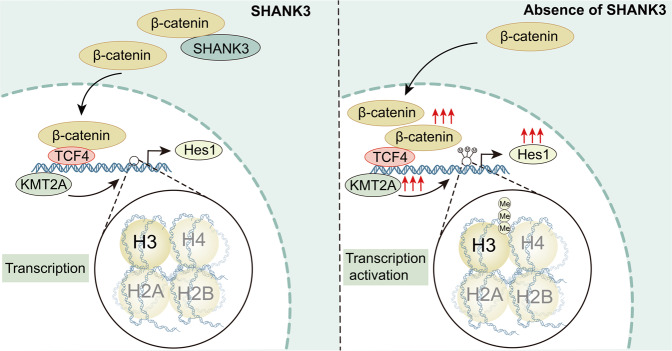
Schematic diagram illustrating the effects of SHANK3 on quiescent NSC activity. SHANK3 regulates KMT2A and Hes1 by mediating the nuclear translocation of β-catenin, thereby affecting the activity of quiescent NSCs.

It is evident that there is a significant decrease in the number of radial glial progenitor cells and immature neuron production in the ventral rather than dorsal dentate gyrus (DG) of the hippocampus in SHANK3-deficient mice (Fig. 6) [11]. Nevertheless, the density of astrocytes and microglia is no significant differences in the ventral and dorsal dentate gyrus of the hippocampus (Fig. 6) [11]. The result points out that SHANK3 governs proliferation and neuronal differentiation in mice ventral dentate gyrus NSCs and impacts functional neurogenesis-related hippocampal behaviors, particularly anxiety-like behaviors and social behaviors [11]. Using adult NSCs from the SVZ of SHANK3 mutant mice, SHANK3 deficiency leads to a decrease in the number of NSCs, neurons, and oligodendroglial cells, whereas an increase in the number of astroglial cells (Fig. 6), thereby leading NSCs to further undergo apoptosis [10]. Interestingly, the absence of SHANK3 does not influence NSC self-renewal capacity while causing morphological fluctuations that result in a propensity to spontaneously adhere despite culture in suspension [10]. The role of SHANK3 as a critical regulator of integrin-dependent processes of cell adhesion, invasion, and migration has been established [16]. Thus, it can be inferred that SHANK3 may play a significant influence on NSC migration, and further investigations are necessary to validate its role in the migration of NSCs in vivo and in vitro. SHANK3 deficiency also affects NSCs and astroglial metabolism, manifesting as endosomal/lysosomal and ubiquitin aggregation together with mitochondrial impairment and inflammasome activation [10]. As illustrated in Fig. 7, in the absence of SHANK3, the LC3B^+^ puncta increases and aggregates, causing ubiquitin aggregation; mitochondrial content decreases, resulting in the formation of autophagosomes. LAMP1^+^ lysosomes exhibit abnormal “clustered” distribution, fusion, and autolysosome formation, accompanied by the activation of inflammatory factors NLRP3 and Caspase1 [10]. Recently, research has authenticated that SHANK3 deficiency decreases the number of total NSCs, active NSCs (aNSCs), and neuroblasts in SVZ and SGZ [49]. In contrast, quiescent NSCs (qNSCs) increased are associated with social deficits due to abnormal epigenetic characteristics, such as histone H3 lysine 4 (H3K4) trimethylation accumulation caused by an increase in KMT2A levels (Fig. 8) [49]. The study further demonstrates that the inhibition of KMT2A and H3K4me3, including molecular and pharmacological inhibition in qNSCs, can restore adult neurogenesis and ameliorate social deficits [49]. These findings offer a novel therapeutic strategy to control qNSC activity as a potential therapeutic target for autism.

Taken together, these discoveries emphasize the importance of SHANK3 in regulating NSC self-renewal, proliferation, differentiation, apoptosis, and metabolism, as well as support the modulation of NSC activity and function as a promising approach to treating neural damage and diseases. It will be interesting to identify inhibitors and activators of the interacting proteins or downstream targets of SHANK in the brain to maintain proper tissue homeostasis, and to clarify the implications of using different drugs. Additional, under normal circumstances, cell-cell interaction in the neurogenic niche is critical for many tasks controlled by the brain, which can be responsible for cellular phenotypes and function. Since cross-talk between cells is often aberrant in diseases, deeper exploration of the regulatory role of SHANK in communication, cross-talk in the neurogenic niche to further understand the integrative capacity of the adult brain, and increase our current knowledge on adult neurogenesis as pharmacological targets or as therapeutics for neurological disorders.

### SHANK and iPSCs

iPSCs are first successfully generated by reprogramming fibroblasts from mice [65]. Presently, advances in iPSC technology have enabled the differentiation of somatic cells into pluripotent stem cells, revolutionizing the ability to study human development and disease. iPSCs can be acquired from human fibroblasts or other somatic cells by the overexpression of specific genes, and these cells can be further differentiated into neural cells, cardiac cells, hepatocytes, myogenic cells, etc. [65–69]. The theoretical potential of using iPSCs as a disease model is evident [69]. Moreover, considering the large number of SHANK variants detected in ASD and/or ID patients so far and the heterogeneity in terms of deletion size identified in PMS patients, iPSCs promise major advances in studying specific SHANK mutations. Although there have been a limited number of studies related to the role of SHANK in supporting the differentiation of iPSCs, recently developed analytic approaches in various fields of molecular biology have led to the discovery of the role of SHANK in the differentiation of iPSCs.

**Fig. 9:**
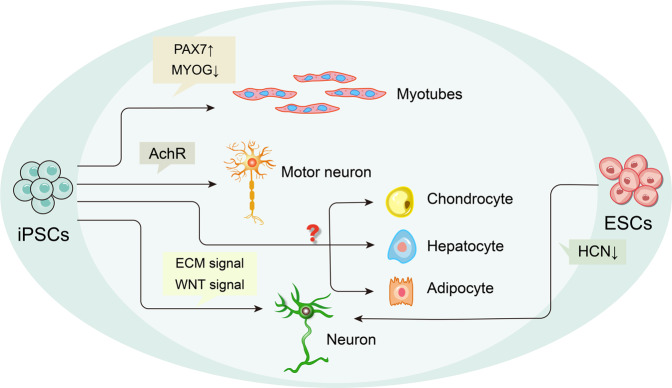
Schematic diagram depicting the effect of the SHANK family on the differentiation of iPSCs/ESCs. Left: The influence of SHANK on iPSC differentiation. Right: The influence of SHANK on neuronal maturation from ESCs.

Transcriptome analyses illustrate that SHANK3 deficiency results in neurogenetic disruption in PMS-derived iPSC-NPCs and may precede alterations in iPSC-neurons [70]. iPSC-neurons have a higher proportion of predicted excitatory neurons than iPSC-NPCs, and conversely, the latter contains a higher proportion of predicted dividing intermediate neuron progenitor cells [70]. This study suggests that SHANK3 plays a vital role in the transcript of iPSC-NPCs and iPSC-neurons and impacts the differentiation of distinct neuronal subtypes, with implications on excitation/inhibition (E/I) balance. Additionally, early developmental pathways are affected, as well as Wnt and ECM signaling in PMS iPSC-NPCs and iPSC-neurons (Fig. 9) [70], providing molecular insights into the management of gene expression by SHANK3. By employing CRISPR/Cas9 nuclease systems to generate SHANK3-deficient iPSCs, an increase in qNSCs and a decrease in aNSCs were observed, which is consistent with the findings of previous murine studies [49]. PMDS-iPSCs can differentiate into slightly fewer neurons, and their excitatory synaptic transmission is impaired, as measured by the expression of GluA1 and GluN1 proteins and the number of Synapsin1^+^ and Homer1^+^/Synapsin1^+^ [71]. Intriguingly, the ability of iPSCs derived from PMDS to form motoneurons is comparable to controls [72]. However, SHANK3 deficiency in motoneurons alters the clustering of postsynaptic acetylcholine receptors (AChRs) in myotubes (Fig. 9) [72]. In addition, SHANK3 mutations cause myogenic cell maturation deficiency, as measured by the expressions of PAX7 and MYOG (Fig. 9) [72]. While SHANK3 can modulate the differentiation and influence the maturation of iPSCs, including neural and myogenic cells, the mechanism underlying its impact on iPSC differentiation into other cell types is still not fully elucidated yet.

iPSCs frequently participate in the maturation of neurons as a cell model. The differentiation process appears to follow the progression of physiological developmental stages, lineage restriction, and neuronal/synaptic maturation, which can be directly observed using appropriate reporting lines, living cell imaging modalities, and single-cell and neural network electrophysiological recordings. For instance, *Shank*3 mRNA could developmentally regulate dendritic localization in iPSC-derived neurons [73]. A significant reduction in the number of Homer puncta in differentiated cortical neurons of PMS patients signals that SHANK3 is essential for Homer1b/c synaptic localization in human neurons [74]. In SHANK2 mutant neurons derived from ASD-affected males, the dendritic length, dendritic complexity, the number of synapses, and frequency of spontaneous excitatory postsynaptic currents are significantly increased [75]. Paradoxically, iPSC-derived neurons infected with shSHANK2 lentivirus demonstrate that SHANK2 deficiency perturbs early neuronal development, including reducing the dendritic length and arborizations of type I vesicular glutamate transporter (VGLUT1^+^) neurons, while increasing cell soma speed of VGLUT1^+^ neurons [76]. Interestingly, knockdowns of SHANK2 in vitro in rodent neurons have typically led to a decrease in synaptic density with a concomitant increase in dendrite complexity [77, 78]. Even though differences exist, they may be due to a species-specific difference in SHANK2 function, cell types, or developmental time points for the phenotypic analysis [75, 76]. These results provide evidence that SHANK3 regulates iPSC differentiation and demonstrate that iPSCs have afforded us exciting opportunities to investigate the role of SHANK in related human disorders. However, current knowledge regarding the regulatory role of SHANK in iPSC differentiation remains fragmented. Further studies are required to define how SHANK regulates cell type- and stage-specific lineage differentiation and the distinctive regulatory mechanisms in iPSCs.

### SHANK and ESCs

ESCs are pluripotent cells derived from the inner cell mass of blastocyst-stage embryos and possess the following unique features: self-renewal and pluripotency [79]. Due to these special properties, ESCs serve as an optimal resource for early embryonic development. For instance, they can be used to explore the impact of specific gene mutations on certain developmental events, enabling the identification of key factors that play a crucial role in cell commitment, differentiation, and reprogramming in adult cells [80]. Despite intense interest, studies about SHANK and ESCs have been scarce to date. PMS-ESCs and ASD-ESCs have proved to be pivotal models for exploring the mechanism of ASD in humans. To specifically analyze the role of SHANK3 in human neuron differentiation and function, heterozygous and homozygous human neurons deficient in SHANK3 are generated in PMS patients. SHANK3 mutated neurons exhibit impaired hyperpolarization-activated cation (I_h_) channels that elicit alterations in neuronal morphology and synaptic connectivity (Fig. 9) [52]. Likewise, neuronal morphogenetic deficits, including cell soma become smaller and neurites increase, had been proved in SHANK3 deficiency ES cell line originating from ASD patients [81]. These results add to the huge body of evidence that ESCs serve as an ideal model to explore the regulatory role of SHANK3 in ESC-differentiated neuronal function. Furthermore, ESCs can reproduce embryogenesis by expressing developmental regulatory genes and activating the molecular pathways through which they occur in vivo. A clear analysis of SHANK in regulating the fate of embryonic embryogenesis is warranted to provide critical insight into therapeutic treatment.

## Conclusion and perspectives

This review briefly illustrated the regulatory role of SHANK in the various behaviors of different stem cell populations. It is paramount to unscramble the mechanisms underlying the functional regulation of SHANK in the fate of multiple stem cells. Unraveling the general profile of complex signaling pathways is also vital, considering this will contribute to a deeper understanding of the function of SHANK and enable the search for novel, unique therapeutic opportunities in diseases. So far, our understanding of SHANK in stem cells remains very much in its infancy, and the role of SHANK in influencing stem cell fate specification and integration is not yet fully fathomed.

Despite considerable progress, much remains to be learned about the regulation of SHANK in stem cells. Our understanding of the direct regulation of stem cell behavior and function by SHANK in stem cells is limited, as are the underlying mechanisms. Future work will undoubtedly explore new roles of SHANK in various stem cell fates, such as cellular adhesion and migration, which are key for neural development, and reveal mechanisms by which SHANK can influence cell function in a comprehensive and systematic manner. In addition, SHANK is essential for carrying out its regulatory role in proliferation, self-renewal, differentiation, apoptosis, and metabolism in NSCs, SCAPs, and iPSCs. However, this has not been determined for other stem cells. For example, SHANK1 and SHANK2 are oncogenes that play a crucial role in tumor development. The mechanism underlying the modulation of behaviors of tumor stem cells by SHANK warrants further exploration. This will be a novel breakthrough in cancer treatment. As described above, SHANK can bind to different proteins through its diverse functional domains. Although it is challenging to list all partners and signaling pathways mediated by the SHANK family, it is evident that our knowledge of the full signal transduction web downstream of the SHANK family is restricted. Future studies can employ high-throughput techniques to map the complete signal transduction network for SHANK in order to comprehend its physiological and pathological role.

SHANK family proteins are very similar in structure but exhibit distinct molecular properties in terms of postsynaptic targeting and assembly [82]. In single-gene mutant mice, residual gene compensation may cover the primary function of SHANK family proteins at glutamate synapses. Simultaneous deletion of SHANK3 and SHANK1 severely impairs postnatal brain development, resulting in morphological and synaptic defects in cortical and hippocampal neurons [82]. Hence, what effect would the deletion of all SHANK family proteins, or two of them, have on stem cell behavior? Alternatively, *Shank1* is one of the fragile X mental retardation protein (FMRP) targets, which are autism risk genes, and altering the translational regulation of *Shank1* transcripts may contribute to the pathology of FXS [83]. Consequently, the impact of deleting the two autism risk genes, as well as their overexpression on brain development and the corresponding compensatory mechanism, warrant further investigation.

Currently, SHANK-based treatments are predominantly aimed at adult restoration of SHANK levels or restoration of downstream mediators [2, 6]. However, few effective drugs are actually available to patients. It is critical to identify novel drug targets and develop new and effective therapies. Utilizing stem cell-targeted therapies optimized via SHANK pathway regulation of downstream targets is likely to expand opportunities for clinical application. Moreover, different protein domains/exons are mutated in SHANK animal models, leading to different phenotypes [31], which should be taken into account when conducting stem cell research to expose these different pathways. Advances in ongoing in vitro and in vivo in stem cell biology will certainly facilitate the discovery of the neurobiological secrets of SHANK proteins, which is likely to fill the gaps and lead to clinical translation toward improved stem cell efficacy.

One overarching certainty for the future is that our knowledge of the biology of SHANK family proteins will keep advancing, and new light will be shed on its effect on stem cell development at the cellular and organismal levels. Finally, based on stem cell therapy, *Shank*-related diseases can be treated with greater confidence in clinics.

## Data Availability

There are no experimental datasets, given that this is a review article that is prepared based on a literature review.
